# Angiography‐Negative Subarachnoid Hemorrhage Detected After Emergent Percutaneous Coronary Intervention for Non‐ST‐Segment Elevation Myocardial Infarction: A Case Report

**DOI:** 10.1002/ccr3.73363

**Published:** 2026-08-23

**Authors:** Chul Hee Lee

**Affiliations:** ^1^ Department of Neurosurgery, College of Medicine Gyeongsang National University Hospital Jinju Republic of Korea

**Keywords:** acute coronary syndrome, angiography‐negative subarachnoid hemorrhage, antiplatelet therapy, hydrocephalus, percutaneous coronary intervention

## Abstract

Neurological deterioration, headache, vomiting, bradycardia, or hypertension after emergent percutaneous coronary intervention should prompt immediate brain imaging. When subarachnoid hemorrhage is detected shortly after coronary stenting, the hemorrhage onset may remain uncertain, and antiplatelet decisions require individualized multidisciplinary assessment.

## 1 Introduction

Subarachnoid hemorrhage (SAH) may cause electrocardiographic abnormalities, arrhythmias, troponin elevation, and stress cardiomyopathy, mimicking acute coronary syndrome (ACS). Conversely, true ACS may present with malignant arrhythmia and cardiac arrest requiring immediate reperfusion and antithrombotic therapy. When ACS and intracranial hemorrhage occur in close temporal proximity, diagnostic prioritization and treatment become difficult, particularly when coronary stenting is followed by detection of high‐grade SAH with hydrocephalus: early dual antiplatelet therapy (DAPT) protects the stent, whereas cerebrospinal fluid (CSF) diversion, intraventricular thrombolysis, and shunt surgery increase hemorrhagic risk.

We report a patient with ventricular fibrillation arrest and non‐ST‐segment elevation myocardial infarction (NSTEMI) from a thrombotic mid‐left anterior descending artery lesion requiring emergent percutaneous coronary intervention (PCI), in whom diffuse angiography‐negative SAH with intraventricular hemorrhage (IVH) and hydrocephalus was detected after PCI. Because no brain imaging was performed before PCI, the exact hemorrhage onset time remained unknown. Existing SAH pathways emphasize early CT, CTA, and catheter angiography [1, 2], but provide little guidance for SAH detected immediately after PCI under potent antithrombotic therapy. This case illustrates the need for urgent neuroimaging, prompt neurosurgical consultation, transparent antithrombotic reporting, and individualized multidisciplinary decision‐making.

## 2 Case History/Examination

**FIGURE 1:**
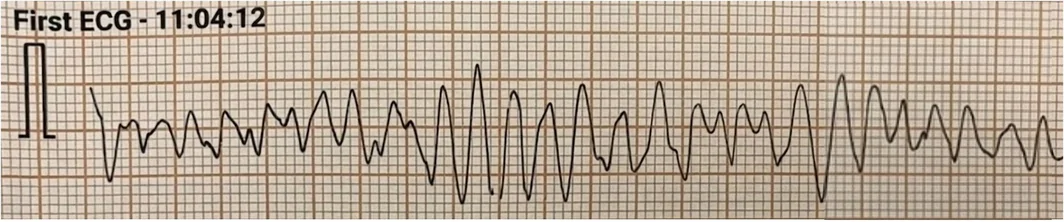
Prehospital rhythm strip documenting ventricular fibrillation as the initial cardiac arrest rhythm. All patient‐identifying information, including calendar date, clock‐time identifiers, patient name, registration number, and emergency medical service identifiers, was removed or masked.

A 64‐year‐old previously independent man collapsed while playing tennis (hospital day 0, 10:56). Bystander CPR was started; EMS arrived at 11:03 and documented ventricular fibrillation (Figure 1). Defibrillation at 11:04 (154 J) achieved transient ROSC before recurrence; a second shock at 11:07 (176 J) achieved sustained ROSC. He reached the emergency department at 11:19.

At 11:20 he was alert, GCS 15, without focal deficit; vital signs were stable except mild tachypnea. He reported chest discomfort, dyspnea, and diaphoresis (NRS 5), treated with morphine. No sedative or paralytic was given before this examination. The first post‐ROSC ECG showed no ST elevation, ST depression in V3/V5, and frequent PVCs. Given chest symptoms, VF arrest, absence of diagnostic ST elevation, a thrombotic culprit lesion, and a rise‐and‐fall troponin pattern, the event was classified as NSTEMI.

**FIGURE 2:**
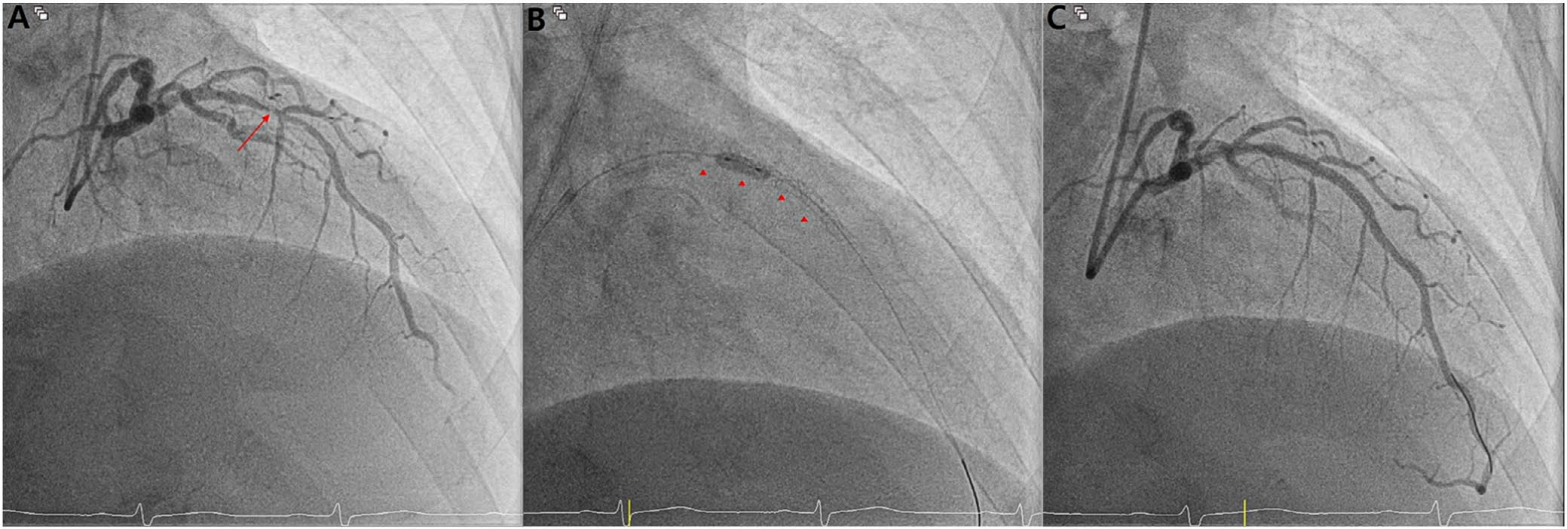
Coronary angiography showing critical thrombotic stenosis of the mid‐left anterior descending artery (A, arrow). Balloon dilatation and stent deployment during percutaneous coronary intervention are shown (B); the red arrowheads indicate the deployed coronary stent. Final angiography demonstrates restoration of coronary blood flow with no residual significant stenosis and Thrombolysis in Myocardial Infarction grade 3 flow (C).

At 11:36, aspirin 300 mg, ticagrelor 180 mg, and rosuvastatin 20 mg were given, with unfractionated heparin (UFH) bolus and infusion. Coronary angiography (12:22) showed a thrombotic 99% mid‐LAD stenosis; PCI with drug‐eluting stent placement was completed at 12:43, restoring TIMI 3 flow with 0% residual stenosis (Figure 2). ACT was not measured. Heparin infusion stopped at 13:07; a single enoxaparin 60 mg dose was given at 15:50 before discontinuation after SAH detection.

### 2.1 Methods: Differential Diagnosis, Investigations and Treatment

Serial troponin T rose from 8 to 1084 ng/L, with a concordant CK‐MB rise, supporting true myocardial infarction. Echocardiography showed preserved ejection fraction (65%) without regional wall motion abnormality.

**FIGURE 3:**
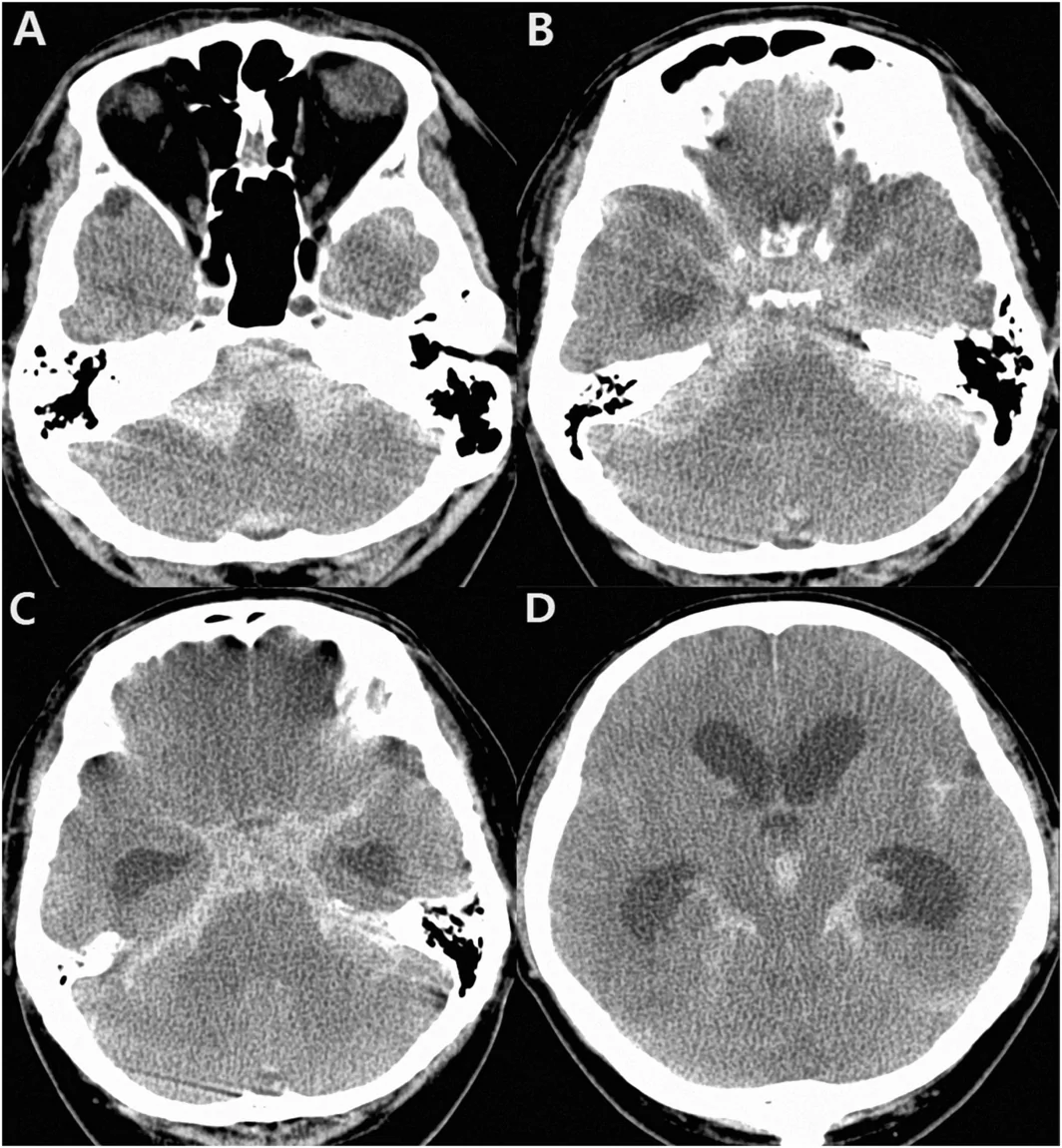
Axial brain computed tomography images obtained after neurological deterioration, with subarachnoid hemorrhage detected after percutaneous coronary intervention, showing diffuse thick subarachnoid hemorrhage centered in the basal cisterns, bilateral Sylvian cisterns, and anterior brainstem cisterns, with intraventricular extension and acute hydrocephalus, consistent with modified Fisher grade 4 subarachnoid hemorrhage (A–D).

From 17:21, the patient developed progressively severe headache (NRS 3 → 8), nausea/vomiting, bradycardia (HR 30–42/min), and hypertension (171/97 mmHg). Despite transient improvement after pethidine, consciousness deteriorated by 21:55 (GCS 5; sluggish pupils). Emergency CT (22:05) revealed diffuse thick SAH in the basal and brainstem cisterns with IVH and acute hydrocephalus (Figure 3) (modified Fisher 4; Hunt‐Hess 5; WFNS 5)—approximately 9 h 22 min after PCI completion. Because no pre‐PCI imaging existed, the exact SAH onset time could not be determined.

At SAH detection, DAPT maintenance dosing had not yet resumed after loading; the last ticagrelor exposure was the loading dose, ~12 h before right EVD placement. Platelet count was 198,000/μL; pre‐PCI coagulation studies were normal. DAPT and anticoagulation were stopped, and single‐donor platelet pheresis (250 mL) was transfused; desmopressin/tranexamic acid were not used.

Given severely impaired consciousness, the patient was intubated and a right frontal EVD placed (23:45, opening pressure > 20 cmH_2_O). Persistent hydrocephalus prompted a left EVD (hospital day 1). Despite bilateral EVDs, thick residual cisternal SAH and hydrocephalus persisted, and lumbar drainage was added (hospital day 2) after CT excluded a mass lesion or herniation risk.

Because dense IVH and cisternal clot persisted despite bilateral EVDs, and CTA/four‐vessel DSA had already excluded a definite ruptured lesion, low‐dose intraventricular alteplase (adapted from the CLEAR III protocol [3]) was administered via the left EVD: 1 mg every 8 h for three doses, with clamping. No recurrent hemorrhage or ventriculitis was observed.

**FIGURE 4:**
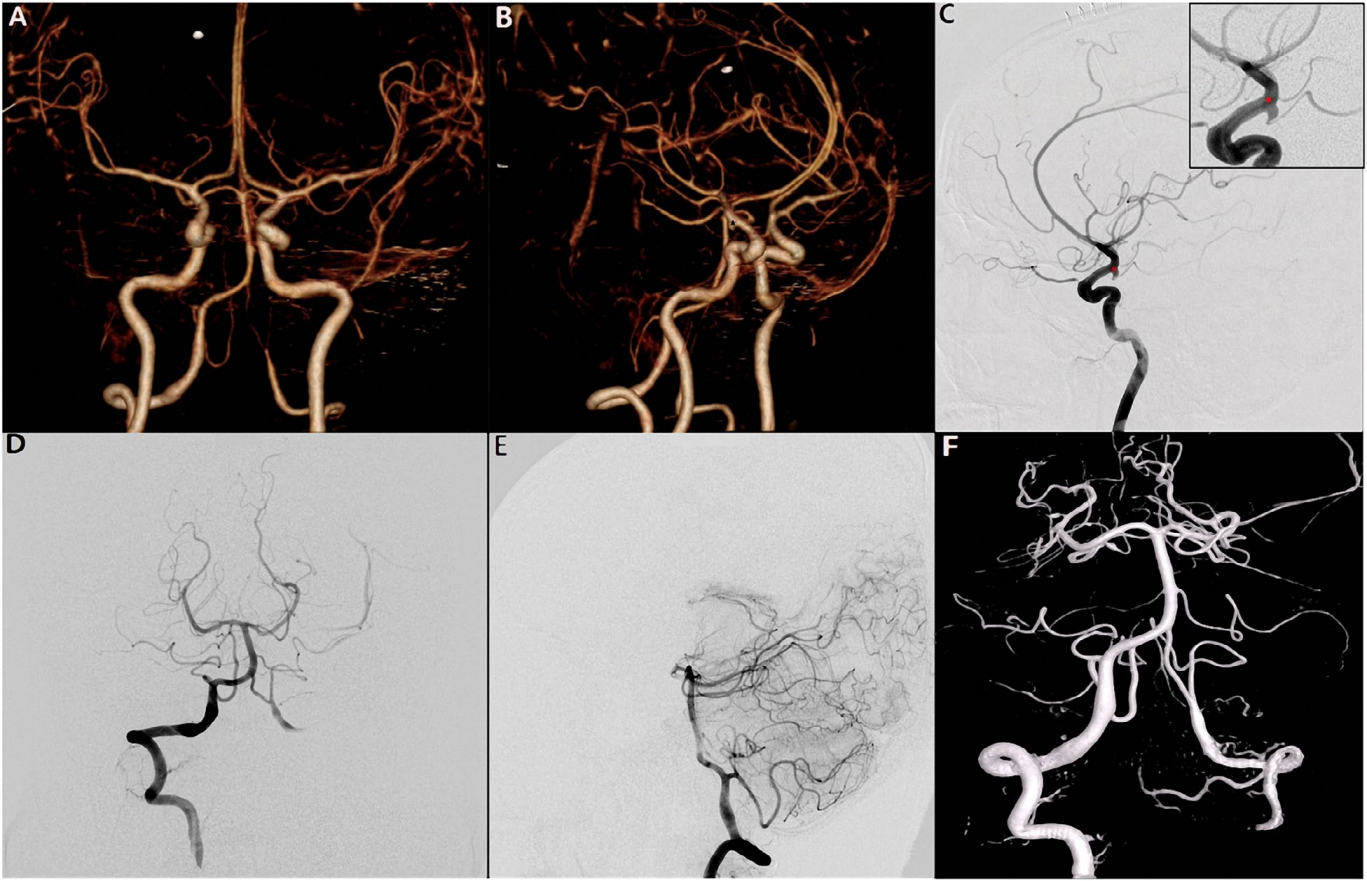
Neurovascular imaging findings after subarachnoid hemorrhage detection. (A) Computed tomography angiography did not identify a definite ruptured aneurysm or other causative vascular lesion. (B) Initial digital subtraction angiography showed no definite aneurysm, dissection, arteriovenous shunt, or other causative vascular abnormality. (C) A small vascular pouch measuring approximately 3 × 3 mm was noted at the right posterior communicating artery origin. The right posterior communicating artery arose from the apex of the pouch, and the lesion had an infundibular configuration without a discrete aneurysmal neck. The magnified inset in the upper right corner highlights the vascular pouch more clearly, supporting the multidisciplinary interpretation of junctional or infundibular dilatation rather than a definite ruptured aneurysm. (D) Right vertebral artery anteroposterior digital subtraction angiography without manual neck compression showed opacification of the contralateral left posterior inferior cerebellar artery. (E) Lateral view of right vertebral artery digital subtraction angiography. (F) Rotational three‐dimensional vertebral artery angiography showed no definite aneurysm, dissection, arteriovenous shunt, or other causative vascular abnormality.

CTA and initial four‐vessel DSA, including vertebrobasilar 3D rotational angiography, revealed no definite aneurysm, dissection, or shunt (Figure 4). A 3 × 3 mm infundibular vascular pouch was noted at the right posterior communicating artery origin, without a discrete neck; after multidisciplinary review, this was interpreted as infundibular dilatation rather than a ruptured aneurysm, and remained stable on follow‐up.

**FIGURE 5:**
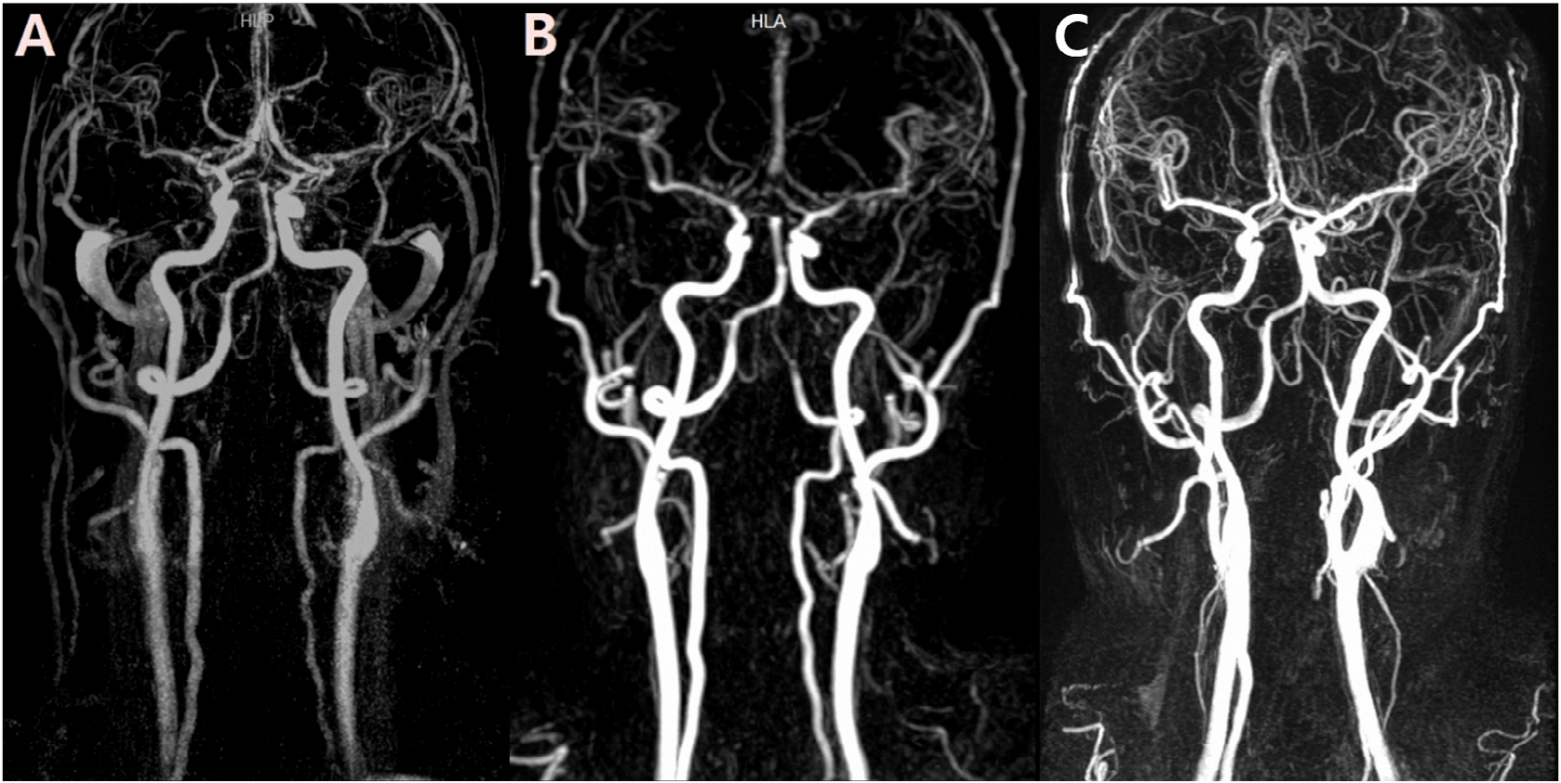
Follow‐up magnetic resonance angiography. Magnetic resonance angiography obtained on hospital day 6 (A) and hospital day 13 (B) showed no definite aneurysm, dissection, or other causative vascular abnormality. Multifocal arterial narrowing compatible with secondary vasospasm was noted on early follow‐up studies. Repeat magnetic resonance angiography obtained more than 5 years after the initial presentation (C) again showed no identifiable causative vascular lesion. These findings support the absence of an identified vascular cause on serial imaging but do not definitively exclude an initially occult ruptured lesion.

Repeat DSA was considered given the diffuse, non‐perimesencephalic pattern, but was deferred because of recent stenting, systemic complications, and antiplatelet‐related bleeding concerns; serial MRA (hospital days 6 and 13, and > 5 years later) was used instead. Serial MRA showed multifocal vasospasm‐compatible narrowing but no causative lesion at any time point (Figure 5), though an initially occult lesion cannot be entirely excluded.

### 2.2 Conclusions and Results: Outcome and Follow‐Up

EVDs were removed on hospital days 3 and 5, and the lumbar drain on day 11 after CT confirmed stable ventricles. Hydrocephalus recurred after drain removal, indicating permanent shunt dependency.

Antiplatelet therapy was individualized through repeated cardiology–neurosurgery discussion: further antiplatelet therapy was withheld immediately after SAH detection; clopidogrel monotherapy was started on hospital day 11 after clinical and imaging stabilization (both EVDs already removed, lumbar drain still in place), then held before VP shunt surgery (day 45; Codman CERTAS valve, opening pressure 27 cmH_2_O), and DAPT (aspirin plus clopidogrel, without ticagrelor) resumed on day 54.

The hospital course was complicated by pneumonia, acute kidney injury requiring CRRT, respiratory failure with tracheostomy, and transient seizure‐like activity responsive to lorazepam. EEG showed diffuse structural dysfunction. A small, clinically insignificant EVD tract hemorrhage occurred; no lumbar drain‐ or shunt‐related hemorrhage, recurrent ICH, stent thrombosis, reinfarction, or recurrent VF were documented. Atrial fibrillation on day 7 resolved with amiodarone.

Functional recovery was gradual: mRS 4 at approximately 3–4 months, improving to 3 by approximately 8 months and 2 by approximately 28 months, remaining 2 beyond 5 years. MMSE was 25/30 at approximately 58 months. At last follow‐up (> 5 years), he was independently ambulatory but had persistent memory/executive deficits and remained shunt dependent. Full chronological, antithrombotic, and neurosurgical details are summarized in Tables 1, 2, 3.

**TABLE 1:** Hospital‐day clock‐time clinical timeline.

Time point	Event/intervention	Key findings/notes
Day 0, 10:56	Collapse during tennis	Sudden collapse during exercise
10:57	EMS call, bystander CPR	CPR initiated
11:03	EMS arrival	Ventricular fibrillation documented
11:04:24	1st defibrillation	154 J; transient ROSC, VF recurred
11:06:39	2nd defibrillation	176 J; sustained ROSC
11:19	ED arrival	Chest discomfort, dyspnea, diaphoresis
11:20	1st neuro exam	GCS 15; no focal deficit
11:20:17	1st 12‐lead ECG	No ST elevation; ST depression V3/V5; PVCs
11:36	DAPT loading + UFH	aspirin 300 mg, ticagrelor 180 mg, UFH bolus/infusion
12:22–12:43	PCI	DES for 99% mid‐LAD stenosis; TIMI 3 flow
13:07	Heparin stopped	UFH infusion discontinued
15:50	Enoxaparin	60 mg SC, single dose
17:21–21:55	Neuro deterioration	Headache→vomiting→bradycardia→GCS 5
22:05	Head CT	Diffuse SAH + IVH + hydrocephalus (modified Fisher 4)
22:09	SAH recognized	DAPT/anticoagulation stopped
23:45	Right EVD	Opening pressure > 20 cmH2O
23:50	Platelet transfusion	250 mL single‐donor pheresis
Day 1	Left EVD, CTA/DSA	No causative lesion; persistent hydrocephalus
Day 2	Lumbar drain, alteplase start	1 mg q8h × 3 via left EVD
Day 3	Right EVD removed	Small tract hemorrhage, not significant
Day 5	Left EVD removed	—
Day 11	LD removed; clopidogrel started	Monotherapy while LD in place
Day 35	Clopidogrel stopped	Pre‐op for VP shunt
Day 45	VP shunt placement	Codman CERTAS, opening pressure 27 cmH2O
Day 54	DAPT resumed	aspirin + clopidogrel
Day 57	Rehab transfer	—
> 3–58 months	Follow‐up	mRS 4 → 3 → 2; MMSE 25/30
> 5 years	Final follow‐up	mRS 2; shunt dependent

Abbreviations: CPR, cardiopulmonary resuscitation; CT, computed tomography; CTA, computed tomography angiography; DAPT, dual antiplatelet therapy; DES, drug‐eluting stent; DSA, digital subtraction angiography; ECG, electrocardiography; ED, emergency department; EMS, emergency medical services; EVD, external ventricular drain; GCS, Glasgow Coma Scale; IVH, intraventricular hemorrhage; LAD, left anterior descending artery; LD, lumbar drain; MMSE, Mini‐Mental State Examination; mRS, modified Rankin Scale; PCI, percutaneous coronary intervention; PVC, premature ventricular complex; ROSC, return of spontaneous circulation; SAH, subarachnoid hemorrhage; SC, subcutaneous; TIMI, Thrombolysis in Myocardial Infarction; UFH, unfractionated heparin; VF, ventricular fibrillation; VP, ventriculoperitoneal.

**TABLE 2:** Antithrombotic exposure and coagulation profile.

Parameter	Value/timing
Aspirin 300 mg	11:36 (loading)
Ticagrelor 180 mg	11:36 (last dose before SAH)
UFH bolus	4000 IU IV at 11:36; +2100 IU at PCI start
UFH infusion	20,000 IU/1 L 5% dextrose in water at 42 mL/h; stopped 13:07
Enoxaparin 60 mg	SC at 15:50; single dose only
ACT during PCI	Not measured
Platelet count	198,000/μL (22:43)
PT/INR/aPTT	11.1 s/0.99/23.7 s (12:31, pre‐PCI)
Fibrinogen	364 mg/dL (hospital day 2)
D‐dimer	4.06 μg/mL
Platelet transfusion	250 mL single‐donor pheresis (23:50)
Desmopressin/TXA	Not used
DAPT interruption duration	Immediate cessation after SAH detection
Clopidogrel monotherapy	Started hospital day 11, 09:00
DAPT resumption	Hospital day 54 (aspirin + clopidogrel)

Abbreviations: ACT, activated clotting time; aPTT, activated partial thromboplastin time; DAPT, dual antiplatelet therapy; INR, international normalized ratio; IV, intravenous; PCI, percutaneous coronary intervention; PT, prothrombin time; SAH, subarachnoid hemorrhage; SC, subcutaneous; TXA, tranexamic acid; UFH, unfractionated heparin.

**TABLE 3:** CSF diversion, intraventricular alteplase, and neurosurgical course.

Procedure	Timing	Notes
Right frontal EVD	Day 0, 23:45	Opening pressure > 20 cmH2O; Kocher point
Left frontal EVD	Day 1	Persistent hydrocephalus/IVH despite right EVD
LD placement	Day 2, 13:40	Added after CT excluded mass/herniation risk
Intraventricular alteplase	Day 2 onward, q8h × 3	1 mg in 1 mL via left EVD; 2 mL flush; clamp 1 h
Right EVD removal	Day 3	Small tract hemorrhage, not clinically significant
Left EVD removal	Day 5	—
LD removal	Day 11	Clamp trial overnight; CT stable; removed 13:50
VP shunt placement	Day 45	Left Kocher point; Codman CERTAS valve, setting 6
Intraoperative CSF pressure	Day 45	27 cmH2O (interpreted cautiously)
Post‐shunt follow‐up CT	Day 51 (6 days post‐op)	No new hemorrhage; stable wound

Abbreviations: CSF, cerebrospinal fluid; CT, computed tomography; EVD, external ventricular drain; LD, lumbar drain; VP, ventriculoperitoneal.

## 3 Discussion

Several features support true ACS as a major contributor—VF arrest, ischemic chest symptoms, a thrombotic culprit lesion, and a clear troponin rise‐and‐fall—yet SAH itself can independently cause ECG changes, arrhythmia, and troponin elevation via catecholamine‐mediated injury [4, 5]. This case therefore illustrates diagnostic uncertainty rather than proof of causality between PCI/antithrombotic therapy and SAH; because no pre‐PCI imaging existed, the true onset of hemorrhage remains unknown.

The key clinical lesson is that new headache, vomiting, bradycardia, hypertension, or delayed neurological recovery after PCI should not be attributed solely to post‐resuscitation status or analgesia, and warrants urgent brain imaging, as occurred here.

Angiography‐negative SAH is heterogeneous; diffuse non‐perimesencephalic patterns with IVH and hydrocephalus, as in this case, carry a higher likelihood of an occult vascular lesion than perimesencephalic hemorrhage [6, 7]. Despite comprehensive initial and long‐term negative vascular imaging, an initially occult ruptured lesion cannot be fully excluded.

Intraventricular alteplase and the antiplatelet strategy were both individualized, risk‐balancing decisions rather than protocolized care. Early DAPT interruption raises stent thrombosis risk [8, 9, 10], while continued DAPT would have increased hemorrhagic risk during CSF diversion and shunt surgery. Existing SAH and ACS guidelines and bleeding‐risk scores (GRACE, CRUSADE, PRECISE‐DAPT, HAS‐BLED) do not specifically address this overlap scenario [11, 12, 13, 14, 15], and prior literature on ICH during DAPT offers limited procedural guidance here [16, 17].

Despite functional improvement, the patient had persistent cognitive deficits and shunt dependence, consistent with known long‐term SAH morbidity [18, 19], supporting structured long‐term multidisciplinary follow‐up in such overlap cases.

This report is limited by its single‐patient design, unknown true SAH onset time, absence of ACT/platelet function testing, deferral of repeat catheter angiography, and lack of transcranial Doppler monitoring. The antiplatelet and alteplase strategies should be regarded as hypothesis‐generating rather than a generalizable pathway.

In conclusion, SAH may be detected in close temporal proximity to emergent PCI, though its true onset may remain uncertain without pre‐PCI imaging. Post‐PCI neurological deterioration warrants urgent brain imaging and neurosurgical consultation, and antithrombotic decisions in such overlap cases require individualized, multidisciplinary judgment.

## 4 Patient Perspective

The patient expressed gratitude for his survival and gradual recovery, noting persistent memory and concentration difficulties that limit complex tasks, while valuing his independent ambulation and ability to engage with family.

## Author Contributions

**Chul Hee Lee:** conceptualization, writing – review and editing, data curation, investigation, writing – original draft.

## Funding

The author has nothing to report.

## Ethics Statement

This study was reviewed by the Institutional Review Board of Gyeongsang National University Hospital, which determined that formal ethics approval was not required for this single‐patient case report (GNUH IRB 2026‐06‐025).

## Consent

Written informed consent for publication of this case report and all accompanying clinical images was obtained directly from the patient prior to submission, after he had recovered sufficient decision‐making capacity. The corresponding author personally explained the contents of the report, the publication process, and the open access nature of the article to the patient in the presence of his spouse.

## Conflicts of Interest

The author declares no conflicts of interest.

## Data Availability

The data supporting the findings of this case report are available from the corresponding author upon reasonable request.
